# Supplementary material for: Synthesis of ZnO/Au Nanocomposite for Antibacterial Applications
Source: Nanomaterials (Basel). 2022 Oct 30;12(21):3832. doi: 10.3390/nano12213832 (PMC9655429; doi:10.3390/nano12213832)
Supplement: Supplementary file 1 [file nanomaterials-12-03832-s001.zip › nanomaterials-1982231-supplementary.pdf]

nanomaterials

Supplementary information

Synthesis of ZnO/Au Nanocomposite for Antibacterial Applications

Violeta Dediu [1,*], Mariana Busila [2], Vasilica Tucureanu [1], Florentina Ionela Bucur [3], Florina Silvia Iliescu [1], Oana Brincoveanu [1] and Ciprian Iliescu [1,4,5,6,*]

1 National Research and Development Institute in Microtechnologies—IMT Bucharest, 126A Erou Iancu Nicolae Street, 077190 Bucharest, Romania
2 Centre of Nanostructures and Functional Materials-CNMF, "Dunarea de Jos" University of Galati, Domneasca Street 111, 800201 Galati, Romania
3 Faculty of Food Science and Engineering, "Dunarea de Jos University" of Galati, Domneasca Street 111, 800201 Galati, Romania
4 Faculty of Chemical Engineering and Biotechnologies, University "Politehnica" of Bucharest, 011061 Bucharest, Romania
5 Academy of Romanian Scientists, 010071 Bucharest, Romania
6 Regional Institute of Oncology, Iasi TRANSCEND Research Center, 2-4 General Henri Mathias Berthelot, Iasi 700483, Romania
* Correspondence: violeta.dediu@imt.ro (V.D.); ciprian.iliescu@imt.ro (C.I.)

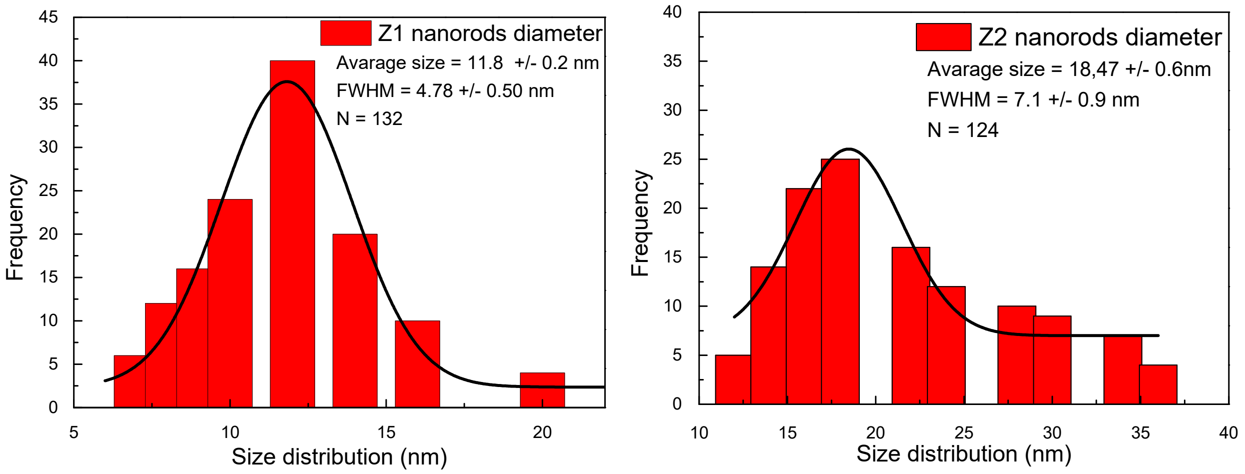

Figure S1. Histograms of ZnO nanorods diameter distribution in sample Z1 (left) and Z2 (right).

The EDX spectroscopy, a semi-quantitative elemental analysis method was used to map the elements in the samples. Multiple spectra were acquired from different areas and the results were averaged.

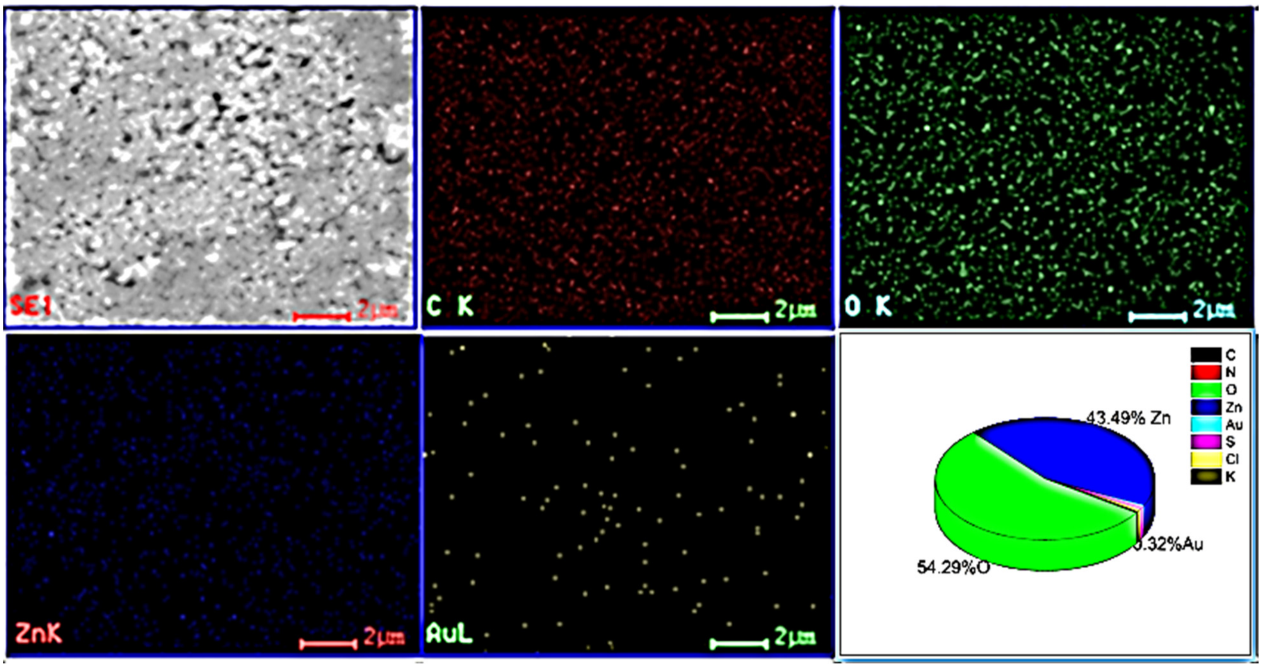

Figure S2. EDX elemental mapping of ZA2 nanocomposite.

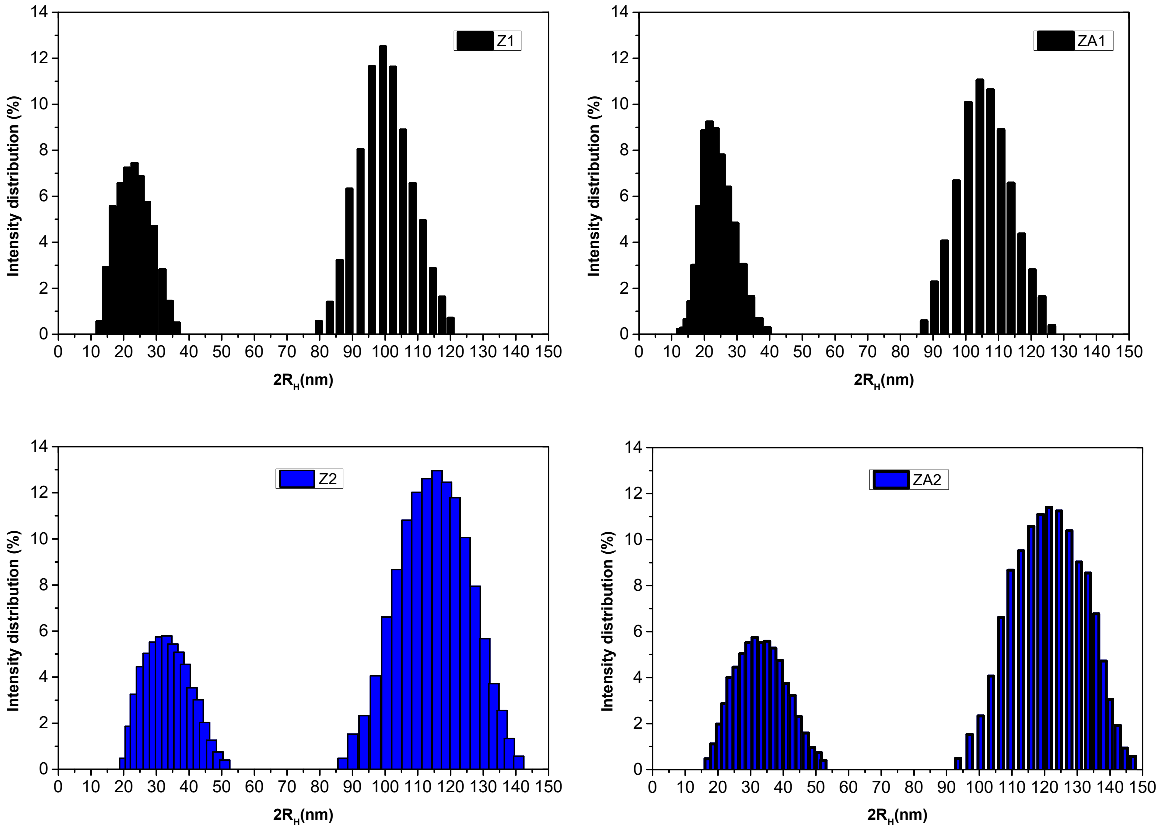

Figure S3. Particles size distribution examined by DLS analysis.

Detection of H_2O_2

Hydrogen peroxide produced by the ZnO and ZnO/Au suspensions was determined through UV-Vis spectrophotometry using KI and soluble starch, applying a method described in [1]. Suspensions of ZnO or ZnO/Au in water (5 mg/mL) were ultrasonicated for 10 min in the dark. Then samples were centrifuged, and 5 mL of each filtrate was transferred to a 10 ml volumetric flask. Different solutions were added successively: 0.5 mL NaCl solution (200 mg/mL), 0.2 mL HCl solution (3.6 vol %), 0.3 mL KI solution (10 mg/mL), and 0.2 mL starch solution (10 mg/mL). The absorbance at 580 nm of resulted solutions was determined using UV–vis spectrophotometry. The generation of H_2O_2 of ZnO and ZnO/Au suspension was determined through reactions with KI and starch, and the results were plotted in Figure S4.

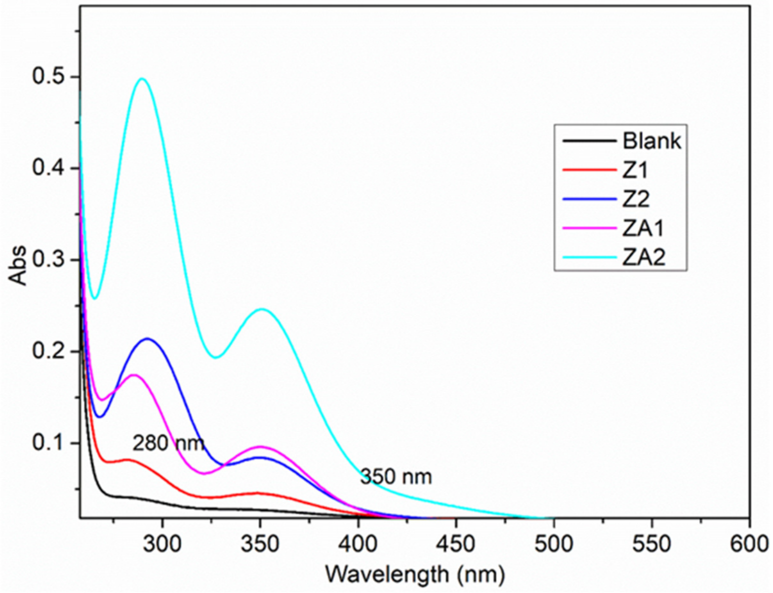

Figure S4. Production of H_2O_2 in the suspensions of ZnO and ZnO/Au samples in the dark.

As shown from absorption spectra from Figure S4, no absorption bands are present around 580 nm in any sample. Two absorption bands at lower wavelengths appeared in all samples, due to the products resulting from the interaction between compounds from the samples and reactants.

References

1. Xu, X., Chen, D., Yi, Z., Jiang, M., Wang, L., Zhou, Z., Fan, X., Wang, Y. and Hui, D. Antimicrobial mechanism based on H_2O_2 generation at oxygen vacancies in ZnO crystals. *Langmuir* **2013**, *29*, 5573–5580.
